# Medical student use of communication elements and association with patient satisfaction: a prospective observational pilot study

**DOI:** 10.1186/s12909-016-0671-8

**Published:** 2016-05-21

**Authors:** Joseph S. Turner, Katie E. Pettit, Bryce B. Buente, Aloysius J. Humbert, Anthony J. Perkins, Jeffrey A. Kline

**Affiliations:** Department of Emergency Medicine, Indiana University, 720 Eskenazi Ave, Indianapolis, IN 46037 USA; Fairbanks School of Public Health, IUPUI, Indianapolis, IN USA

**Keywords:** Scripted communication, Patient satisfaction, Medical education

## Abstract

**Background:**

Effective communication with patients impacts clinical outcome and patient satisfaction. We measure the rate at which medical students use six targeted communication elements with patients and association of element use with patient satisfaction.

**Methods:**

Participants included fourth year medical students enrolled in an emergency medicine clerkship. A trained observer measured use of six communication elements: acknowledging the patient by name, introducing themselves by name, identifying their role, explaining the care plan, explaining that multiple providers would see the patient, and providing an estimated duration of time in the emergency department. The observer then conducted a survey of patient satisfaction with the medical student encounter.

**Results:**

A total of 246 encounters were documented among forty medical student participants. For the six communication elements evaluated, in 61 % of encounters medical students acknowledged the patient, in 91 % they introduced themselves, in 58 % they identified their role as a student, in 64 % they explained the care plan, in 80 % they explained that another provider would see the patient, and in only 6 % they provided an estimated duration of care. Only 1 encounter (0.4 %) contained all six elements. Patients’ likelihood to refer a loved one to that ED was increased when students acknowledged the patient and described that other providers would be involved in patient care (*P* = 0.016 and 0.015 respectively, Chi Square). Likewise, patients’ likelihood to return to the ED was increased when students described their role in patient care (*P* = 0.035, Chi Square).

**Conclusions:**

This pilot study demonstrates that medical students infrequently use all targeted communication elements. When they did use certain elements, patient satisfaction increased. These data imply potential benefit to additional training for students in patient communication.

**Electronic supplementary material:**

The online version of this article (doi:10.1186/s12909-016-0671-8) contains supplementary material, which is available to authorized users.

## Background

Communication skills have profound implications for healthcare providers. Effective communication can improve patient outcomes and reduce malpractice liability [1–3]. It is also associated with patient satisfaction [4], which has become increasingly emphasized throughout hospital systems. Recently, patient satisfaction scores have had an increasing effect on physician reimbursement.

Despite the salient importance of communication, further research is needed to clarify the best methods of teaching this skill to medical providers. While much research has been done in this area in recent years, systematic review and meta-analyses of existing patient-centered communication education trials have produced mixed results, with only 40 % reporting a positive outcome [5]. Notably, none of the studies included in this systematic review were set in the emergency department, highlighting the lack of quality emergency medicine research in this area. Furthermore, as the authors of the review note, undergraduate medical students are also underrepresented in communication education literature, with only one study in this review using them as participants.

Medical schools have traditionally underemphasized communication as a testable skill [6, 7]. In recent years, however, research in this area has increased and we have learned more about effective methods for improving communication skills in medical students [8, 9]. As a result, attempts have been made to refine and expand communication education programs in undergraduate medical curricula [10, 11]. Some schools have adopted communication into their competency based curricula to encourage development in this area [12]. These programs aim to teach students to overcome communication challenges such as dealing with an angry patient, delivering bad news, and addressing end of life care.

Such programs could potentially improve measurable, patient-oriented aspects of the interaction between patient and provider including patient satisfaction and outcomes. However, before focusing on these higher-level skills, medical educators may want to test if students employ the most basic elements of provider-patient communication and what effect this has on patient satisfaction.

Scripted communication offers one simple methodology educators may use to improve basic communication. In recent years, commercial consulting agencies and hospital administration have collectively promulgated scripted communication when interacting with patients as a way to increase patient satisfaction. The Studer Group’s AIDET℠ mnemonic is one example of a strategy to promote scripted communication. The mnemonic reminds the provider to perform such basic tasks as acknowledging the patient by name, introducing themselves by name, and explaining the steps in the patient’s care plan. However, evidence of scripted communication education improving patient satisfaction remains relatively scarce in the medical literature. The study authors, working with a medical librarian, conducted a MEDLINE search to locate articles specifically focused on the effect scripted communication has on patient satisfaction. Two small studies from the radiology literature report an increase in patient satisfaction after radiology technicians implemented scripted communication [13, 14]. One emergency department study indicated that scripted communication at triage may decrease elopement rates [15]. Only one small study has mentioned scripting education for medical students [16]. Thus, the magnitude of the benefit of scripted communication for medical students remains largely unquantified.

The objectives of this study were to measure the association of six basic elements of communication with patient satisfaction in the emergency department and to establish a baseline rate at which medical students use these communication elements as a pilot for a larger interventional trial.

## Methods

### Design

We conducted a prospective observational study in the emergency departments of two academic, urban hospitals, each with over 100,000 patient visits annually. The study was conducted between June 2013 and August 2013 as a pilot study for an upcoming interventional trial.

### Participants

Participants included volunteer fourth year medical students participating in the required emergency medicine clerkship. All students at Indiana University School of Medicine participate in a brief session introducing scripted communication prior to their third year. They did not receive any additional training for this pilot study. At the beginning of the course, medical students were given a brief informational session about participating in a study to assess skills in the emergency department, but were otherwise blinded to the nature of the study.

Patients who could provide verbal consent (>18 y/o or had a parent present to consent) in English or Spanish and who were evaluated by a participating medical student were given the option to participate in a patient satisfaction survey. We did not administer surveys to patients with the following conditions: under arrest, altered mental status, a psychiatric chief complaint (suicidal ideation, homicidal ideation, aggressive behavior, depression, anxiety, or psychosis), or critical illness (unstable vital signs, respiratory distress, or triaged to the high acuity area of our emergency departments).

### Key outcome measures

We measured two main outcomes: rate of use of six targeted communication elements by the student and association between the use of the communication elements and results of a patient satisfaction survey. Six elements of communication were chosen by the study investigators prior to the study (Table 1). These elements were chosen because they are already taught to resident and faculty physicians in the participating emergency departments, they are closely related to components of existing scripting tools such as the AIDET℠ tool created by the Studer Group®, and they could be accurately observed during the initial student-patient encounter.

**Table 1:** Observed communication elements

1) Did the student acknowledge the patient using the patient’s name?
2) Did the student introduce himself/herself by name?
3) Did the student explain his/her role as a medical student?
4) Did the student explain some of the steps (including diagnostic testing, medication administration, or observation) that would be used to address the patient’s complaint?
5) Did the student explain that additional providers (such as a resident or attending physician) would also be evaluating the patient?
6) Did the student offer an estimated duration of time that the patient would spend in the ED?^a^

^a^For estimated duration, a general statement of time (e.g. “overnight” or “a few hours”) was considered acceptable; a specific number was not required

The patient satisfaction survey had four components: 1) a question asking if the interaction with the medical student made the patient more likely to choose that ED in the future,2) a question asking if the interaction with the medical student made the patient more likely to refer a loved one to that ED, 3) a question asking patients to rate the student’s overall communication skill on a 5-point Likert scale, and 4) a modified version of a previously validated Communication Assessment Tool (CAT) (Table 2). The CAT tool assesses interpersonal and communication skills using a 15-item survey with a 5-point Likert scale (1 = poor, 2 = fair, 3 = good, 4 = very good, 5 = excellent) [17, 18]. The survey was modified by removing one question, “The doctor’s staff treated me with respect”, to keep focus on the student-patient interaction rather than the patient’s overall experience.

**Table 2:** Patient satisfaction survey

A. Does your interaction with the medical student make you more likely to choose this emergency department in the future? Y N
B. Does your interaction with the medical student make you more likely to refer a friend or loved one to this emergency department? Y N
C. How would you rate the student’s overall communication skills?
Poor 1 2 3 4 5 Excellent
D. How well did the medical student do in the following areas:^a^
Poor 1 2 3 4 5 Excellent
1. Greeted me in a way that made me feel comfortable
2. Treated me with respect
3. Showed interest in my ideas about my health
4. Understood my main health concerns
5. Paid attention to me
6. Let me talk without interruptions
7. Gave me as much information as I wanted
8. Talked in terms I could understand
9. Checked to be sure I understood everything
10. Encouraged me to ask questions
11. Involved me in decisions as much as I wanted
12. Discussed next steps, including any follow-up plans
13. Showed care and concern
14. Spent the right amount of time with me

^a^Modified from Mercer et al. Patient perspectives on communication with the medical team: Pilot study using the Communication Assessment Tool-Team (CAT-T). *Patient Education and Counseling*, 73(2), 220–223

### Procedure

A Masters of Public Health student who received training on communication and navigation of the emergency department from the study investigators served as an observer and recorded data from student-patient encounters by following the students and directly observing the encounters in real-time. This data included whether or not the student utilized each of the six communication elements as well as whether the student performed 17 additional “dummy” data points. Refer to Additional file 1 for the complete data collection sheet with all “dummy” data points. These data points were not outcomes, but were chosen by study investigators as actions commonly performed by students in the emergency department that may be of interest to a clerkship director and were meant to keep the student blind to what elements were of interest to the observer. For the targeted communication elements, the observer made no judgment about the effectiveness of the communication; he simply recorded a positive response if the student made any attempt to utilize the communication element. For example, a student telling the patient in general that some tests would be ordered was considered a positive response for element #4, regardless of whether the student knew the appropriate tests to order, thus maintaining the focus on communication rather than medical knowledge or other aspects of patient care.

Following the student-patient encounter, prior to discharge or admission, the observer returned to the patient’s room and orally administered the patient satisfaction survey, using an interpreter when necessary for Spanish speaking patients. During the survey, the observer presented the patient with a picture of the student and stressed that the questions applied specifically to the patient’s interaction with that student and not other aspects of the patient’s care in the ED. The satisfaction survey was done without the students’ knowledge. The data collected was entered in Microsoft Excel for further analysis.

### Data analysis

Descriptive analytics were performed using SAS 9.3 to determine the frequency of individual and combined script use. For each of the six communication elements, the patient was assigned to one of two groups depending on whether or not the patient was exposed to the communication element (independent variable). Each of the six communication elements was coded dichotomously. The group designation was determined based on whether the observer witnessed the student verbally utilizing the communication element. Analysis was performed to establish association of each of the six primary communication elements with each of the four components of the patient satisfaction survey. The outcome (dependent variable) was either a response of “yes” to the likelihood to return or likelihood to refer questions, a score of “5” (excellent) for overall communication skill, and overall score on the CAT. The first 3 outcomes (likelihood to return, likelihood to refer, excellent overall communication) are dichotomous measures, while the fourth outcome (overall score on the CAT) is a continuous measure.

We tested for the bivariate association of communication elements with likelihood to return, likelihood to refer, and excellent overall communication skill using a Chi-square test (*P* < 0.05 significant). In addition we used mixed effects logistic regression to test the association of communication elements with these three outcomes while adjusting for other characteristics. In this model a random effect for student was included to account for association within each student. Fixed effects included the communication element of interest, ED site, student age, and student gender.

Since the overall CAT score was continuous, we used the Wilcoxon rank sum to test whether the overall CAT score differed across the communication elements. A mixed effect linear regression model was used to test whether CAT scores differed by communication elements adjusting for ED site, age, and gender.

For each outcome, the mixed effect *p*-values were adjusted for multiple comparisons using the Adaptive Hochberg assessment.

## Results

Forty-one medical students were approached for consent. One medical student opted out of the study, so forty medical students were observed during the three-month study period. Twenty-seven (67.5 %) were male. Thirty (75 %) planned to pursue emergency medicine and ten (25 %) planned to pursue other specialties (including internal medicine, obstetrics-gynecology, anesthesiology, and “unsure”). Two hundred sixty-one medical student-patient interactions were observed. Data for five observations were incomplete and ten patients refused the survey. These data were excluded, leaving 246 complete data sets for analysis.

**Fig. 1:**
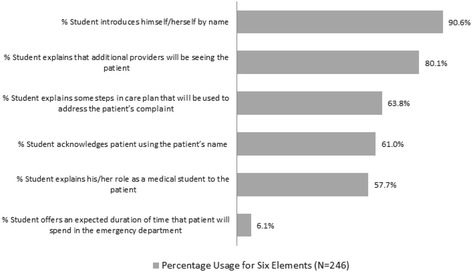
Communication element use

Data for use of the communication elements is shown in Fig. 1. The most often used element was the student introducing himself or herself by name, which happened in 90.6 % (223/246) of encounters. The least commonly utilized element was providing the patient with an expected duration of stay, which occurred during 6.1 % (15/246) of encounters. Only one encounter (0.4 %) included all six communication elements. Forty-four percent of encounters included three or fewer elements, and the vast majority of encounters (81.7 %) included four or fewer elements.

Table 3 shows the percentage of communication element use across patient satisfaction. The student explaining their role as a medical student was significantly associated with a patient’s likelihood to choose the emergency department again for the unadjusted (*P* = 0.035) and adjusted analyses (*P* = 0.038). The student acknowledging the patient by name and the student explaining that other providers would be seeing the patient were significantly associated with a patient being likely to refer loved ones to the emergency department in the bivariate analysis (*P* = 0.016 and 0.015 respectively); and in the mixed effects logistic regression models (*P* = 0.014 and 0.024). The student acknowledging the patient by name was associated with the patient giving the student an excellent (5 on a 5-point Likert scale) rating for overall communication for the unadjusted analysis (*P* = 0.011) and the adjusted analysis (*P* = 0.009). Explaining the steps in the patient’s care plan was associated with an excellent communication rating in the unadjusted analysis (*P* = 0.038) but did not reach statistical significance after adjusting for ED site, age, and gender (*P* = 0.067).

**Table 3:** Association of element use with patient satisfaction (*N* = 246)

	% Student encounter would make choose ED again	% Student encounter would make refer loved one to ED	% Rate student’s overall communication skill = 5 (Excellent)
Student did not acknowledge patient by name (*n* = 96)	89.6	81.2	64.6
Student acknowledged patient by name (*n* = 150)	90.7	92.0	80.0
*P*-value	0.780 (0.821)	0.016 (0.014)*	0.011 (0.009)*
Student did not introduce himself/herself by name (*n* = 23)	95.6	87.0	78.3
Student introduced himself/herself by name (*n* = 223)	89.7	87.8	73.5
*P*-value	0.359 (0.390)	0.902 (0.958)	0.623 (0.616)
Student did not describe his/her role as a medical student (*n* = 104)	85.6	84.6	76.9
Student described his/her role as a medical student (*n* = 142)	93.7	90.1	71.8
*P*-value	0.035 (0.038)*	0.198 (0.306)	0.369 (0.298)
Student did not explain any steps in care plan (*n* = 89)	91.0	84.3	66.3
Student explained some steps in care plan (*n* = 157)	89.8	89.7	78.3
*P*-value	0.760 (0.775)	0.209 (0.133)	0.038 (0.067)*
Student did not explain that other providers would see patient (*n* = 49)	85.7	77.6	63.3
Student explained that other providers would see patient (*n* = 147)	91.4	90.3	76.6
*P*-value	0.232 (0.191)	*0.015* (0.024)*	0.056 (0.057)
Student did not provide an estimated duration of time for ED stay (*n* = 231)	90.9	87.0	73.6
Student provided an estimated duration of time for ED stay (*n* = 15)	80.0	100.0	80.0
*P*-value	0.167 (0.186)	0.135 (0.133)	0.584 (0.610)

*Statistical significance defined as a *P*-value < 0.05. The first *P*-value in each set is derived from Chi Square analysis. The second *P*-value (in parentheses) is derived from mix effects logistic regression model. Values that are underline/italicized remained significant after multiple comparisons

Table 4 displays the mean number of questions on the modified Communication Assessment Tool in which the student scored “excellent” compared with communication element use. The following elements were statistically associated with an increased performance as measured by a higher number of “excellent” ratings on the tool: acknowledging the patient by name (*P* = 0.028 unadjusted; *P* = 0.034 adjusted) and explaining that other providers would be evaluating the patient (*P* = 0.027 unadjusted; *P* = 0.012 adjusted). Giving an estimated duration of stay was associated with a higher CAT score in the unadjusted analysis (*P* = 0.041) but did not reach statistical significance in the adjusted analysis (*P* = 0.097).

**Table 4:** Association of use with performance on CAT

	Mean (SD) # CAT questions rated at 5 (Excellent)
Student did not acknowledge patient by name (*n* = 96)	11.0 (3.8)
Student acknowledged patient by name (*n* = 150)	12.0 (3.3)
*P*-value	0.028 (0.034)*
Student did not introduce himself/herself by name (*n* = 23)	12.0 (3.1)
Student introduced himself/herself by name (*n* = 223)	11.5 (3.6)
*P*-value	0.543 (0.588)
Student did not describe his/her role as a medical student (*n* = 104)	11.6 (3.7)
Student described his/her role as a medical student (*n* = 142)	11.5 (3.4)
*P*-value	0.356 (0.828)
Student did not explain any steps in care plan (*n* = 89)	11.3 (3.4)
Student explained some steps in care plan (*n* = 157)	11.7 (3.6)
*P*-value	0.116 (0.400)
Student did not explain that other providers would see patient (*n* = 49)	10.5 (3.9)
Student explained that other providers would see patient (*n* = 147)	11.8 (3.4)
*P*-value	0.027 *(0.012*)*
Student did not provide an estimated duration of time for ED stay (*n* = 231)	11.5 (3.6)
Student provided an estimated duration of time for ED stay (*n* = 15)	13.0 (2.3)
*P*-value	0.041 (0.097)*

*Statistical significance defined as a *p*-value < 0.05. The first *P*-value in each set is derived from Chi Square analysis. The second *P*-value (in parentheses) is derived from mix effects linear regression model. Values that are underline/italicized remained significant after multiple comparisons

After adjusting for multiple comparisons the following associations remained significant: acknowledging the patient by name-likelihood to refer (adjusted and unadjusted analyses); acknowledging the patient by name-overall satisfaction (adjusted and unadjusted analyses); explaining that other providers would see the patient-likelihood to refer (unadjusted only); and explaining that other providers would see the patient-CAT performance (adjusted only).

## Discussion

This study indicates that medical students use targeted communication elements inconsistently, though these elements may have an important effect on patient satisfaction. Although communication is recognized as a critical skill for healthcare providers, medical school education in communication has been underemphasized [6, 7]. Scripting has been strongly advocated across many disciplines and levels of training as a basic communication education strategy that may benefit learners and patients. As relatively inexperienced communicators practicing in a difficult environment, medical students rotating in the emergency department may be in a position to benefit the most from this strategy. Prior to implementing a scripting-based strategy designed to improve professional competence, we believe it is important to measure current communication behaviors of medical students used on real patients, and the satisfaction scores from these same patients.

Our study represents a rare look at student behavior during actual encounters in an emergency department setting. We chose six elements of provider-patient communication which are currently stressed to residents and faculty at our institutions and which are closely related to components of commonly utilized scripting tools. Some of these communication elements, such as acknowledging the patient by name, may seem like obligatory parts of any professional interaction between provider and patient. However, we discovered that medical students use these targeted communication elements inconsistently. Based on anecdotal reports and observation prior to the study, we had hypothesized that medical students would frequently acknowledge the patient by name and introduce themselves by name, but rarely explain the next steps in the patient’s care plan or provide an estimated time to completion of the care plan. Our expectations for the student failing to explain the care plan or provide an estimated time to completion of the care plan were confirmed. The fact that the students only acknowledged the patient by name 61 % of the time was a surprising result and suggests that educators cannot assume that these basic elements of communication are indeed occurring during medical student and patient interactions. Further research and education may therefore be warranted.

This study also gathered baseline data about patient satisfaction with medical student care and evaluated whether there was an association between use of key communication elements and patient satisfaction. We used a number of a number of outcome measures to assess patient satisfaction. We chose likelihood to return or refer a loved one as primary outcome measures because these are routinely measured in patient satisfaction surveys in the United States and have significant effects on physician reimbursement. We also incorporated the CAT tool as a measure of patient satisfaction because it has been well-validated in a practice setting similar to ours [18]. Studies assessing other measures of patient satisfaction may be helpful for other practice settings.

Baseline patient satisfaction with medical student encounters in our study was surprisingly high, with 90 % of the patients indicating they were more likely to return and 88 % saying they were likely to refer a loved one based on their encounter with the student. We predicted a lower baseline for patient satisfaction.

Despite the high baseline satisfaction, these data indicate a positive association between use of the communication elements and patient satisfaction. Four outcomes of interest were correlated with each of the six communication elements, resulting in 24 comparisons. For 17 of these 24 comparisons there was a trend toward increased patient satisfaction being associated with use of the communication element. Eight of these comparisons reached statistical significance in the Chi Square analysis and six were statistically significance after adjusting for other factors using a mixed effects model. However, these results must be approached with caution; after adjusting for multiple comparisons, only 3 comparisons for each model remained significant. These comparisons were split among 2 communication elements: acknowledging patient by name and explaining that other providers would be seeing the patient.

These data alone do not prove that use of these communication elements improves patient satisfaction. Most comparisons in our study did not reach statistical significance, possibly because the number of encounters was too small, and association alone does not indicate causation. Nevertheless, the trend toward improved satisfaction indicates additional study is warranted. If a true association does exist, the inconsistency with which our students use these elements could be a cause for concern and it may indicate that further emphasis on incorporating the scripting of key communication elements should be a priority in medical education. We may find, as this study suggests, that certain elements of scripted communication are more important than others and this is where our educational focus should be concentrated.

This was a pilot study looking at student communication behaviors during actual patient encounters and the association between use of certain communication elements and patient satisfaction. We are currently conducting a more extensive study in which medical students will receive formal training in scripting as a means of improving communication skills. Additional data from this study should further clarify the value of scripting education in these providers and in this setting. We do not know how this would translate to other care settings, such as the ward or clinic, or with more seasoned providers, such as residents and faculty. Future research in this area would be valuable.

### Limitations

There were several limitations to this study. The study group consisted of a small sample of medical students from a single medical school. A large percentage (75 %) of the students observed were seeking to pursue a career in Emergency Medicine. Additionally, 67.5 % of the medical students were male as compared to 52 % in graduating medical school classes nationwide per AAMC 2011 data. It is unknown whether these factors would result in a baseline increase or decrease in communication element use. While we stressed to the patient that the survey pertained only to their encounter with the student, it is possible that other aspects of their visit including interactions with other providers and perception of time influenced survey results. It is also likely that other verbal and even non-verbal communication elements that were not measured influenced the results [19]. Therefore, causality cannot be determined from this association. It is also possible that the baseline frequency of medical student use of the chosen communication elements was artificially increased due to the Hawthorne effect. While we took extensive measures to keep students blind to the nature of the study, the presence of an observer may still have influenced student behavior during the encounters.

Finally, our sample size may have been too small given the multiple comparisons adjustment. Post-hoc calculations revealed less than 40 % power to detect 10 % absolute difference in outcomes.

## Conclusions

To our knowledge, this is the first study to evaluate the use of targeted communication elements by medical students during actual emergency department patient encounters. In this pilot study, we found that medical students do not routinely use communication elements during student-patient interactions. We also found that there is a trend towards increased patient satisfaction when these communication elements were used. Further study is needed to quantify how effective scripting education amongst medical students could be in improving patient satisfaction.

## Additional file

Additional file 1: Data collection sheet. (DOCX 15 kb)

## Abbreviations

CAT: communication assessment tool
ED: emergency department
