# Supplementary material for: Medical student use of communication elements and association with patient satisfaction: a prospective observational pilot study
Source: BMC Med Educ. 2016 May 21;16:150. doi: 10.1186/s12909-016-0671-8 (PMC4875631; doi:10.1186/s12909-016-0671-8)
Supplement: Additional file 1: — Data collection sheet. (DOCX 15 kb) [file 12909_2016_671_MOESM1_ESM.docx]

**Appendix A – Data Collection Sheet**

1. Student Name ____________
2. Student ID# ____________
3. ED Site where student is rotating _____________
4. Time Initial Observation Begins____________
5. Time Initial Observation Ends_____________
6. Time of Survey____________

**Observation Data Points**

1. Did the student wash their hands before entering the room?

Y N

1. Did the student acknowledge the patient using the patient’s name?

Y N

1. Did the student introduce himself/herself by name?

Y N

1. Did the student explain his/her role as a medical student?

Y N

1. Did the student ask any specific details about the patient’s complaint?

Y N

1. Did the student ask about the patient’s medications?

Y N

1. Did the student ask about the patient’s allergies?

Y N

1. Did the student ask about past surgeries?

Y N

1. Did the student ask if the patient uses tobacco?

Y N

1. Did the student ask how much alcohol the patient drinks?

Y N

1. Did the student listen to the patient’s lungs?

Y N

1. Did the student palpate the patient’s abdomen?

Y N

1. Did the student look in the patient’s throat?

Y N

1. Did the student look in the patient’s ears?

Y N

1. Did the student explain some of the steps (including diagnostic testing, medication administration, or observation) that would be used to address the patient’s complaint?

Y N

1. Did the student explain that additional providers (such as a resident or attending physician) would also be evaluating the patient?

Y N

1. Did the student offer an estimated duration of time that the patient would spend in the ED?

Y N

1. Did the student thank the patient for coming in?

Y N

1. Did the student order any tests prior to staffing with the attending physician?

Y N

1. Did the student order any medications prior to staffing with the attending physician?

Y N

1. Did the student perform any procedures on the patient?

Y N

1. Did the student call any consults on the patient?

Y N

1. Did the student go back and talk to the patient prior to discharge or admittance?

Y N
